# Rare presentation of lateral meniscus tear with pathognomonic MRI finding

**DOI:** 10.1016/j.ijscr.2019.11.025

**Published:** 2019-11-19

**Authors:** Mohamed Al Dosari, Aissam Elmhiregh, Mohamed Hammad, Sayed Alam, Shamsi Hameed

**Affiliations:** Hamad Medical Corporation, Hamad General Hospital, Al Rayan Road, Doha, P.O Box 3050, Qatar

**Keywords:** Meniscus tear, Popliteus tendon, Magnetic resonance, Meniscectomy.

## Abstract

**Introduction:**

Meniscal tears are common knee injuries that occur in different populations and particularly among athletes. Either isolated or accompanied with anterior cruciate ligament injury, meniscal tears can be a source of significant knee pain, locking and instability.

Different patterns of meniscal tears are described radiologically and arthroscopically, however displacement of the torn part of the meniscus can cause characteristic appearance on MRI.

**Presentation of the case:**

The reported case is of a 27-year-old, previously healthy gentleman who presented to our care with acute sharp left knee pain and infrequent locking symptoms after playing football. The patient was limping and had lateral joint line tenderness along with positive McMurray’s test for lateral meniscus. MRI study showed double popliteus tendon sign on the sagittal cuts. The patient was operated on elective list and underwent lateral meniscus posterior flap tear partial meniscectomy as the tear was in the white zone.

On follow up after 4 months, the patient was satisfied and had regained full pre-injury functional capacity.

**Conclusion:**

Displaced lateral meniscus tear into the popliteal hiatus can be seen as a characteristic double popliteal sign in MRI as the displaced meniscus flap runs on the tibial surface parallel to the popliteus tendon.

## Highlights

• Sport injury led to lateral meniscal posterior horn flap tear.
• This flap tear displaced posteriorly in our reported patient in the popliteus hiatus.
• This displaced tear appeared as double popliteus tendon in the sagittal MRI.
• The patient underwent arthroscopic partial meniscectomy and recovered very well in the later follow ups.

## 1 Introduction

Meniscal tears are a common knee injury that can be of variable presentations, causes, symptoms and radiological appearance [1,2]. Knee twisting injuries during sports is a common cause of meniscal tears in general and variable shapes and configurations of meniscal tears have been described. A mean annual incidence of 66 per 100,000 has been reported [3,4].

Knee pain is a cardinal symptom of meniscal tears, other symptoms like clicking and locking can also occur especially in displaced flap or bucket handle tears. Menisci is an important knee structure that play a crucial role in load transmission, lubrication and joint stability [5,6]. Hence, meniscal injuries can lead to significant knee disability and requires treatment in most of the case.

**Fig. 1:**
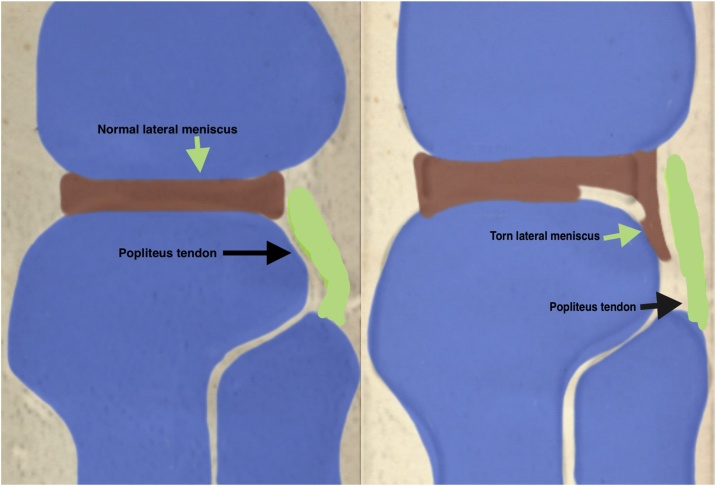
Illustration of displaced flap tear of posterior horn of lateral meniscus to mimic double popliteus tendon.

Beside clinical examination, magnetic resonance plays an important role in diagnosing meniscal tears and the patterns of the tear can be visualized by radiologists in most of the cases. Tears appears as T2 high intensity signal reaching the meniscal surface in more than one MRI cut. Displaced meniscal flaps or bucket handles can be seen in different sequences and can be seen in different places in the joint. Several radiological patterns have been described as the double PCL sign in case of displaced Bucket handle tear of medial meniscus and double popliteus tendon sign in cases of posterior displacement of posterior flap tear of lateral meniscus over the superior posterior aspect of the lateral tibia through the popliteus hiatus to run parallel to popliteus tendon (Fig. 1).

This is a case report of a patient with lateral meniscal tear with MRI evidence of double popliteus sign that was written in line with SCARE criteria of case reports [10].

## 2 Presentation of the case

This is a case report of 27-year-old gentlemen, who was previously healthy, non-smoker and with no positive family history of chronic illnesses. The patient was presented to our care after referral from primary health center with acute on chronic left knee pain especially with ambulation and frequent knee locking. The patient is working as a clerk and playing football as a hobby 3–4 times per week. He reported having a fall on his left knee 1 year prior to presentation, that led to knee pain which subsided afterwards. The patient resumed playing football despite his knee pain and did not seek medical care until he sustained twisting knee injury one month prior to presentation to our care. The pain later increased and he had more frequent episodes of locking.

**Fig. 2:**
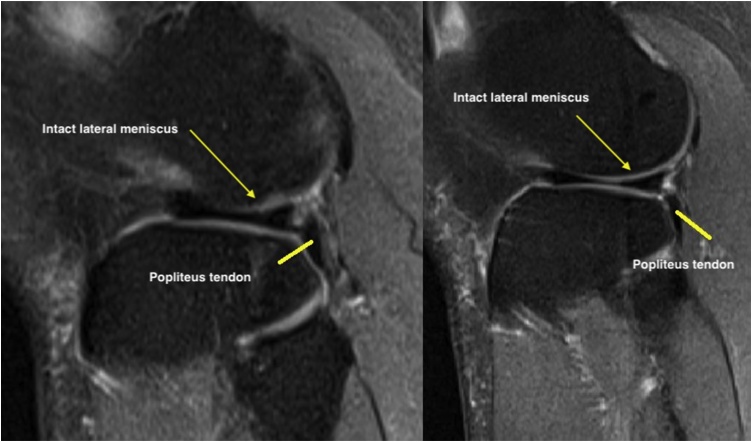
Sagittal T2 MRI cuts showing Normal MRI anatomy.

**Fig. 3:**
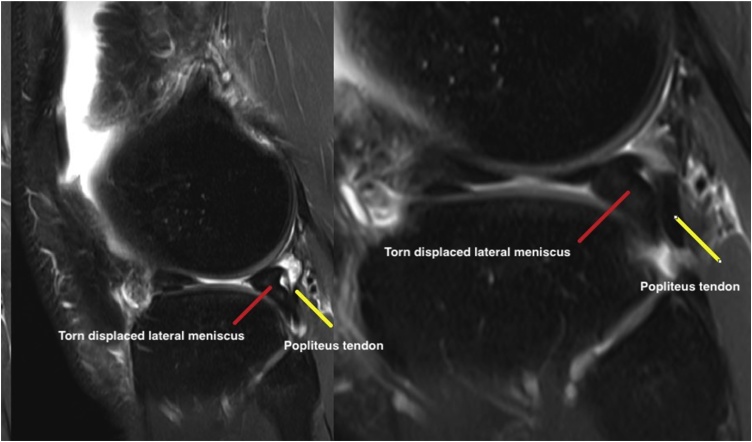
Sagittal T2 MRI cuts showing Double popliteus sign.

**Fig. 4:**
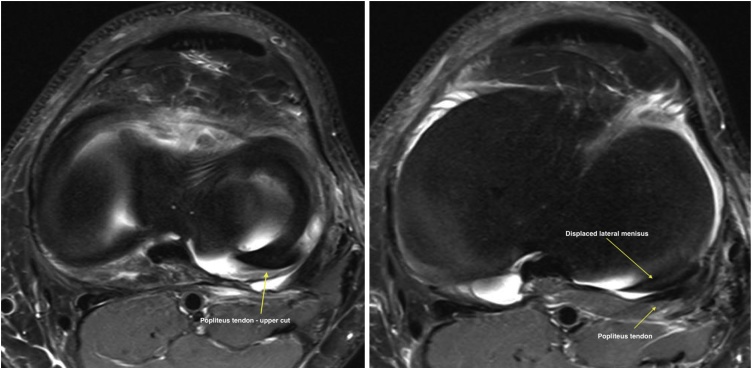
Axial T2 cuts showing axial illustration of displaced posterior horn of lateral meniscus.

His knee examination was remarkable for limited range of motion from 0 to 90 degrees only, mild knee effusion and positive McMurray test for lateral meniscus. Lachman, Drawers, pivot shift and dial tests were all negative. While plane radiographs of the knee did not show a knee pathology, magnetic resonance study was reported by a senior radiology consultant as demonstrating a flap tear of the undersurface of the body of the lateral meniscus, which was displaced posteriorly in the popliteus hiatus as a double popliteus tendon sign (Fig. 2, Fig. 3, Fig. 4).

**Fig. 5:**
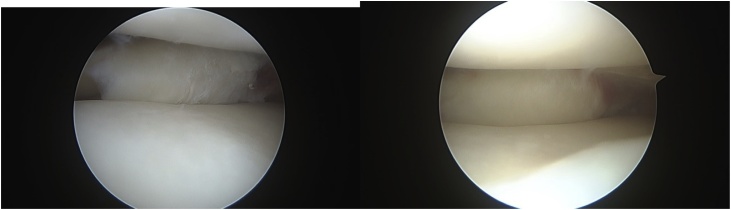
Arthroscopic pictures intraoperatively showing the displaced lateral meniscus flap.

**Fig. 6:**
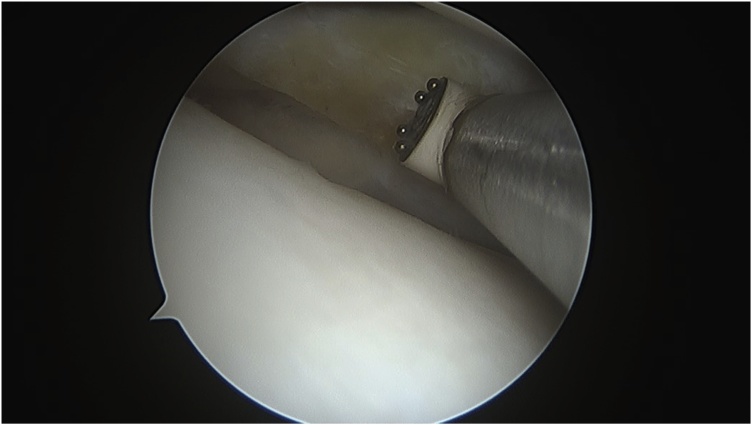
Arthroscopic picture intraoperatively showing final treatment after partial meniscectomy.

After detailed discussion and counseling of the patient and his family, the patient was booked electively for arthroscopic meniscal tear reduction with repair versus partial meniscectomy. The surgery was done by a senior orthopedic consultant under general anesthesia and the arthroscopic finding confirmed the diagnosis of displaced flap tear of the body of the lateral meniscus in the popliteus hiatus. The flap was reduced but it was beyond repair as the tear was in the white zone of the meniscus. Hence partial meniscectomy was done with balancing of the meniscus. The rest of knee was normal (Fig. 5, Fig. 6).

The patient was discharged the same day from the hospital in good general condition. His surgical wounds healed without complication and he started full weight and range of motion has improved remarkably achieving full range by the 4^th^ postoperative week. His knee pain ceased by the 8^th^ week and he returned to sport by the 12^th^ postoperative week.

## 3 Discussion

Literature search revealed that double popliteal tendon sign has been reported once [7]. In that case, a complex flap tear was displaced in the popliteus hiatus and underwent partial meniscectomy. Meniscus in general plays a crucial role in the biomechanics of the knee [8] and any pathology or the absence of a meniscus can significantly affect knee function. Hence the aim of our treatment was to preserve as much meniscal tissue as possible after reduction of the displaced flap. Since repair was not achievable due to presence of the tear in the avascular zone of the meniscus, limited partial meniscectomy with balancing of the edges was undertaken.

As no meniscal repair was done, accelerated knee rehabilitation protocol with early full weight bearing and unrestricted range of motion was initiated [9]. The patient was allowed to return to sports after his postoperative knee pain diminished and as soon as he regained good quadriceps and hamstring control. The absence of any concomitant knee injury facilitated good and quick recovery.

Magnetic resonance imaging interpretation plays an important role in planning surgical treatment of meniscal pathology as there are significant changes of the meniscal tissue and mechanical characteristics after surgical treatment [6]. Meniscectomy usually lead to some mechanical load transmission changes across the joint and hence degenerative changes will be a sequel of major unplanned meniscectomy [11]. In our case, MRI showed the size and the extent of the meniscal flap and limited partial meniscectomy was planned aiming for best short- and long-term outcomes.

## 4 Conclusion

Displaced lateral meniscus tear into the popliteal hiatus can be seen as a characteristic double popliteal sign in MRI as the displaced meniscus flap runs on the tibial surface parallel to the popliteus tendon.

## Sources of funding

This paper is self-funded by the authors with no sponsorship.

## Ethical approval

This study has been approved by the medical research center in the state of Qatar and the patient of the reported finding has been consented for publication.

## Consent

The patient with reported finding has been consented before writing the case report and publication.

## Author contribution

Dr. Mohamed Al Dosari … study concept, data analysis or interpretation.

Dr. Aissam Elmhiregh … data collection, data analysis or interpretation, writing the paper.

Dr. Mohamed Hammad … writing the paper.

Dr. Sayed Alam … data analysis or interpretation.

Dr. Shamsi Hameed … data analysis or interpretation.

## Registration of research studies

researchregistry5128.

## Guarantor

Dr. Aissam Elmhiregh.

## Provenance and peer review

Not commissioned, externally peer-reviewed

## Declaration of Competing Interest

All Authors declare no conflict of interest neither employment, consultancies, stock ownership, honoraria, paid expert testimony, patent applications/registrations, nor grants or other funding.
